# Recognition of high-specificity hERG K+ channel inhibitor-induced arrhythmia in cardiomyocytes by automated template matching

**DOI:** 10.1038/s41378-021-00251-4

**Published:** 2021-03-16

**Authors:** Hao Wang, Hongbo Li, Xinwei Wei, Tao Zhang, Yuting Xiang, Jiaru Fang, Peiran Wu, Xi Xie, Ping Wang, Ning Hu

**Affiliations:** 1 The First Affiliated Hospital of Sun Yat-sen University, School of Electronics and Information Technology, State Key Laboratory of Optoelectronic Materials and Technologies, Guangdong Province Key Laboratory of Display Material and Technology, Sun Yat-sen University, Guangzhou, 510006 China; 2 Biosensor National Special Laboratory, Key Laboratory of Biomedical Engineering of Ministry of Education, Department of Biomedical Engineering, Zhejiang University, Hangzhou, 310027 China; 3 The Sixth Affiliated Hospital of Sun Yat-sen University, Guangzhou, 510655 China; 4 State Key Laboratory of Transducer Technology, Chinese Academy of Sciences, Shanghai, 200050 China

**Keywords:** Electrical and electronic engineering, Chemistry

## Abstract

Cardiovascular disease (CVD) is the number one cause of death in humans. Arrhythmia induced by gene mutations, heart disease, or hERG K^+^ channel inhibitors is a serious CVD that can lead to sudden death or heart failure. Conventional cardiomyocyte-based biosensors can record extracellular potentials and mechanical beating signals. However, parameter extraction and examination by the naked eye are the traditional methods for analyzing arrhythmic beats, and it is difficult to achieve automated and efficient arrhythmic recognition with these methods. In this work, we developed a unique automated template matching (ATM) cardiomyocyte beating model to achieve arrhythmic recognition at the single beat level with an interdigitated electrode impedance detection system. The ATM model was established based on a rhythmic template with a data length that was dynamically adjusted to match the data length of the target beat by spline interpolation. The performance of the ATM model under long-term astemizole, droperidol, and sertindole treatment at different doses was determined. The results indicated that the ATM model based on a random rhythmic template of a signal segment obtained after astemizole treatment presented a higher recognition accuracy (100% for astemizole treatment and 99.14% for droperidol and sertindole treatment) than the ATM model based on arrhythmic multitemplates. We believe this highly specific ATM method based on a cardiomyocyte beating model has the potential to be used for arrhythmia screening in the fields of cardiology and pharmacology.

## Introduction

In past decades, with changes in human diet and lifestyle, the incidence of cardiovascular diseases (CVDs) has risen year by year. According to statistical data released by the World Health Organization (WHO), CVDs cause 17.9 million deaths worldwide each year, and CVD-related deaths exceed cancer-related deaths, accounting for 31% of all disease-related deaths^1^. CVDs, which can instantly result in death, have become the number one cause of death in humans. In China, nearly 4 million people die suddenly due to CVDs every year, accounting for over 40% of the total annual deaths^2^. It is estimated that approximately one patient dies of CVD every 10 s. Even if the most advanced treatments are applied in a timely manner, more than half of patients lose their self-care ability due to CVD^3,4^. It is worth noting that CVDs affect both elderly people and younger people, and this has been especially true in recent years. Arrhythmia is an important type of CVD that can be induced by gene mutation, heart disease, or pathobolism^5^. EADs and DADs are important signs of arrhythmia and drug-induced arrhythmia. EADs occur in phases 2 and 3 of the action potential, causing multiple cardiomyocyte contractions in a single period. In addition, DADs occur in phase 4 of the action potential and cause further depolarization after repolarization. Arrhythmia may occur alone or in conjunction with other CVDs and can cause sudden death or lead to heart failure due to the continuous involvement of the heart^6–8^. Moreover, drug-induced arrhythmia threatens the health of patients, is responsible for the withdrawal of approved drugs and wastes a large number of resources. For example, inhibition of the human ether-à-go-go-related gene (hERG) potassium channel by drugs induces prolongation of repolarization and even prolonged QT syndrome in some severe cases, negatively affecting normal cardiac beating^9^. The ability of drugs to block hERG channels has been reported. Various cardiovascular and noncardiovascular drugs can inhibit hERG channels, and careless use of these drugs causes serious consequences. Therefore, it is necessary to establish an effective model or platform for investigating the mechanism of arrhythmia.

Conventional heart models used to study arrhythmia include living animal models, heart tissue models, and cultured cardiomyocytes^10–14^. Electrocardiographs (ECGs) can be recorded from living animals or heart tissues to predict arrhythmia^15,16^. A large number of methods for high-throughput arrhythmic recognition, including optical video analysis, multiwell patch-clamp recording, microelectrode arrays (MEAs), and interdigital electrodes (IDEs), have been proposed, as shown in Table 1. In detail, optical video analysis can be used to analyze cell beating, movement, and action potentials and obtain information about intracellular Ca^2+^ levels based on cell video analysis with high-speed imaging system^17–22^. Long-term cellular monitoring can be achieved using label-free optical video analysis^23^. However, to achieve higher identification accuracy and obtain special cellular information, fluorescent dyes have been widely used in experiments. For example, voltage-sensitive dyes have been used for intracellular action potential monitoring, while calcium fluorescent dye can be used to obtain information about intracellular Ca^2+^ levels^24,25^. In addition, fluorescence assays such as fluorescent imaging plate reader (FLIPR)-based assays, can obtain high-quality images with high resolution and contrast, which can improve the analysis of cell beating and movements^17,19^. However, the adverse effects of fluorescent dyes may affect cell status, limiting long-term monitoring ability. Furthermore, optical video analysis is difficult to perform in real time because it requires massive computing resources. Representing an improvement over the gold standard technique, multiwell patch-clamp recording can be applied to study arrhythmia in a high-throughput manner. However, patch-clamp recording is invasive and cannot be used for long-term monitoring^26,27^. To overcome this limitation, MEAs, IDEs, and cantilevers were introduced to record the extracellular potentials or mechanical beating signals of cultured cardiomyocytes in a long-term, dye-free, and noninvasive manner^28–34^. However, arrhythmic signals are conventionally analyzed by parameter extraction or examination by the naked eye in these cases, making it difficult to achieve automated and efficient arrhythmia recognition. In this study, automated template matching (ATM) is proposed as a method for real-time long-term recognition of arrhythmia at the single beat level with low computational complexity. Dye-free and noninvasive ATM is performed with an interdigitated electrodes (IDEs) impedance detection system. Moreover, as the quantitative analysis is a major goal in the study of arrhythmias, we aim to use our detection system for quantitative analysis^35^.

**Table 1:** The common methods to analysis the arrhythmic

Method	Label and invasion	Protocol	Efficient	Throughput	Long-term detection	Accuracy	Real-time	Ref.
Multi-well patch clamp	Yes	Complicated	Low	High	No	High	Yes	^26,39^
Traditional MEAs and IDEs methods	No	Simple	Low	High	Yes	High	Yes	^28,34^
Optical video analysis without dye	No	Complicated	High	High	Yes	Low	No	^23,40^
Optical video analysis with dyes	No	Complicated	High	High	No	High	No	^41,42^
Automated template matched method	No	Simple	High	High	Yes	High	Yes	This work

**Fig. 1:**
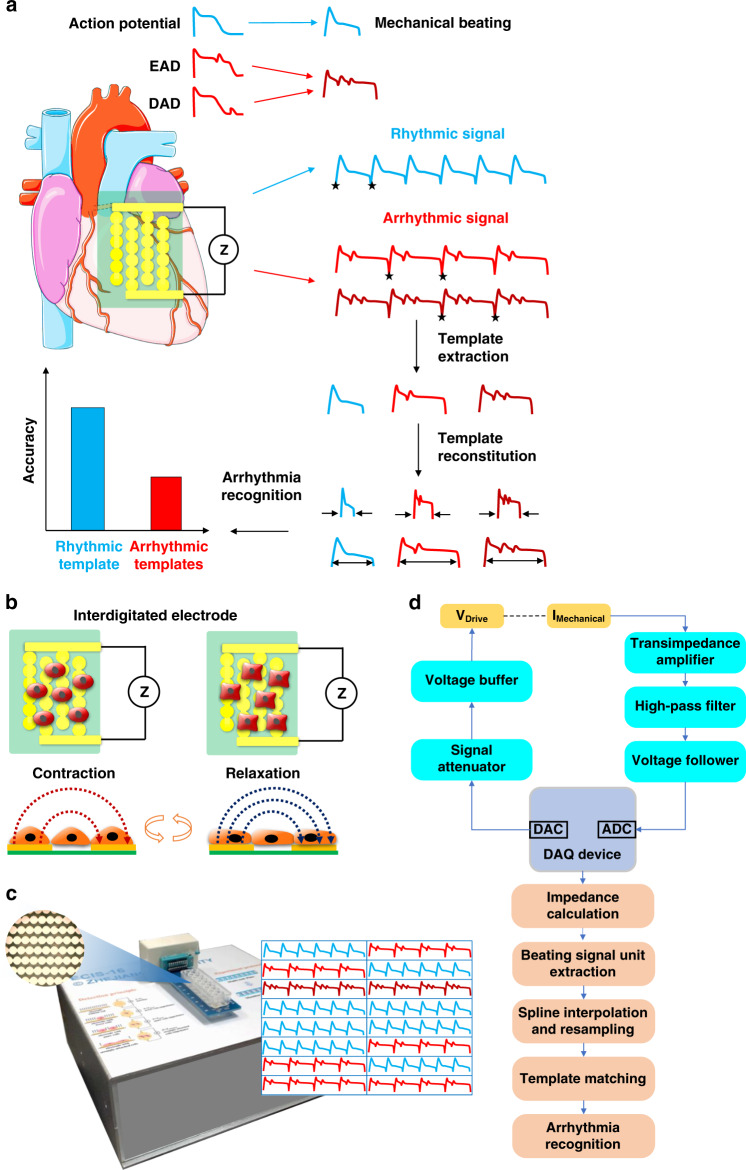
High-specificity recognition of hERG K^+^ channel inhibitor-induced arrhythmia in cardiomyocytes by automated template matching. **a** Schematic of cardiomyocyte arrhythmia recognition by automated template matching for mechanical beating signals. The cardiomyocyte biosensing system can record rhythmic and arrhythmic mechanical beating signals from cardiomyocytes by interdigitated electrode (IDEs) and an impedance measurement technique. The rhythmic and arrhythmic templates are randomly extracted by feature point determination and data collection. These templates are then reconstituted by spline interpolation to match the data length of the targeted signals. Arrhythmia recognition is finally performed by calculating the correlation between the templates and targeted signals. **b** Working principle of mechanical beating recording by interdigitated electrodes (IDEs) and an impedance measurement technique. The rhythmic mechanical beating signals are relevant to the rhythmic contraction and relaxation of cardiomyocytes, which affect the ion current by changing cell morphology, cell-cell attachment, and cell-electrode attachment. **c** Images of the automated template matching (ATM) biosensing system for automated cardiomyocyte mechanical beating signal collection and analysis with the multiwell IDE device. ATM is capable of continuous and parallel monitoring of many samples. **d** Block diagram of the automated template matching (ATM) biosensing system including hardware and software modules. The hardware components include a signal generation module (orange), analog signal conditioning module (cyan), and an integrated data acquisition (DAQ) module (blue) with digital-to-analog convertor (DAC) and analog-to-digital convertor (ADC) functions. The software components include automated signal acquisition, processing, and analyzing modules (red)

Here, we develop a unique ATM cardiomyocyte beating model with an IDE impedance measurement system (Fig. 1a). Based on the detection principle of IDEs, the rhythmic beating behavior of cultured human induced pluripotent stem cell-derived cardiomyocytes (iPSC-CMs) can be measured in the form of rhythmic impedance beating signals. Native rhythmic signals and drug-induced arrhythmic signals can both be recorded by the cardiomyocyte biosensing system. To establish the ATM model, templates were extracted from rhythmic and arrhythmic beating signals and were subsequently reconstituted by spline interpolation to dynamically match the data length of the target signals. To characterize the performance of the ATM model, the rhythmic template and arrhythmic multitemplates obtained after astemizole treatment were individually tested under long-term treatment with astemizole at different doses, droperidol, sertindole, and E-4031. The rhythmic template presented a higher recognition accuracy than the arrhythmic multitemplates. Moreover, the ATM model can be applied for other techniques, such as optical image analysis and MEAs. We believe this new ATM model will provide a promising and alternative method for arrhythmia screening for cardiological and pharmaceutical applications.

## Experimental methods

### Device fabrication

A borosilicate glass slide (Corning, USA) was cut into 80 mm × 15 mm pieces and used as the substrate of a 2 × 8 IDE array. IDE tracks were fabricated by photolithography, deposition, and liftoff processes. In detail, the glass slide was cleaned with acetone, isopropanol, and deionized water and dehydrated on a 200 °C hotplate for 10 min. Then, a Microposit S1813 photoresist (Rohm and Haas, USA) was spincoated on a glass slide at 3000 rpm, prebaked for 60 s at 115 °C, exposed to 20 mW/cm^2^ I-line (365 nm) for 2.5 s, and developed in a Microposit MF CD-26 developer (Shipley, USA) for 40 s. Subsequently, 10 nm Ti/100 nm Au was deposited on the pattern slide by a thermal evaporator, liftoff was performed in acetone, and the slide was rinsed with isopropanol and deionized water. As shown in Fig. 1b, interdigitated branches were patterned with a 90-µm diameter circle-on-line. The center-to-center spacing of the adjacent branches was 120 μm. A 2 × 8 biocompatible polystyrene-based multiwell with a diameter of 5 mm was finally fixed on the IDE device for cell culture.

### Detection principle of cardiomyocyte mechanical beating

The mechanical beating of cardiomyocytes was detected by IDEs and an impedance measurement technique. IDEs are conventionally driven by a low-amplitude sinusoidal signal with a fixed working frequency, and the ion current generates and flows between IDE pairs. When IDEs are covered with seeded cells, the ion current will be affected by cell attachment, spreading, and proliferation, which can be reflected by the changes in the measured impedance. Based on this detection principle, the mechanical beating of cardiomyocytes, which can induce weak impedance fluctuations due to changes in cell morphology, cell-cell attachment, and cell-substrate attachment, can also be detected by IDEs. The measured impedance of IDEs presents rhythmic changes due to the rhythmic mechanical beating of cardiomyocytes (Fig. 1b). Therefore, the mechanical beating of cardiomyocytes can be detected by measuring IDE impedance.

### Development of the ATM biosensing system

The ATM biosensing system is a multichannel recording instrument that uses a multiwell IDE device to collect and analyze the mechanical beating signals of cultured cardiomyocytes (Fig. 1c). The ATM biosensing system contains hardware and software modules. The main functions of the system are signal recording and ATM. Linear correlation analysis of the data and template is the key strategy used for ATM, and a larger correlation indicates more similar profiles between the data and template. In this study, the linear correlation between two variables was calculated by the correlation coefficient. The formula is as follows:

1 $$r\left( {X,Y} \right) = \frac{{{\mathrm{Cov}}\left( {{X},{Y}} \right)}}{{\sqrt {\mathrm{Var}}\left[ X \right]{\mathrm{Var}\left[ Y \right]} }}$$

where Cov(*X*, *Y*) is the covariance of *X* and *Y*, Var[*X*] is the variance of *X*, and Var[*Y*] is the variance of *Y*. *r* < 0 means *X* is negatively correlated with *Y* and *r* > 0 means *X* is positively correlated with *Y*. For mechanical beating signal acquisition, a sinusoidal drive signal of frequency 10 kHz and amplitude 30 mV was generated to drive the IDE device via a digital-to-analog converter (DAC) module on data acquisition (DAQ) device, signal attenuator, and voltage buffer. The sinusoidal current signal at the same frequency was converted and amplified to a voltage signal by the transimpedance amplifier. The AC voltage signal was then processed by a high-pass filter and a voltage follower and recorded by an analog-to-digital (ADC) module on the DAQ device. Subsequently, signal processing was carried out by a customized LabVIEW program. Cardiomyocyte mechanical beating signals were initially calculated by fast Fourier transform (FFT) with a data output interval of 12.8 ms. The signals were denoised by 5-point smoothing filtering to reduce the noise interference when extracting the feature points. To improve the temporal resolution of signals, spline interpolation was applied to improve the signal resolution to 1.28 ms to accurately extract feature points and calculate the correlation. The beating signal unit was extracted by valley detection by the adaptive threshold algorithm. The templates of mechanical beating were initially extracted and stored in the program, which was further processed by spline interpolation and resampling to dynamically and precisely match the data length of the recorded beating signal units and analyze the correlation between the templates and each beating signal unit to complete the ATM. A correlation threshold was set to assess the arrhythmia. Moreover, the eye observation method was used to calculate the recognition accuracy of ATM.

### Cardiomyocyte culture

The device was sterilized in 70% ethanol (Sigma-Aldrich, USA) under UV exposure in a biosafety cabinet for 2 h and then coated with 50 μl of 10 mg/ml fibronectin (Sigma-Aldrich, USA) solution in Ca^2+^/Mg^2+^-free phosphate-buffered solution (PBS, (Sigma-Aldrich, USA)) inside an incubator at 37 °C, 5.0% CO_2_ for 2 h to improve cell adhesion. Human-iPSC-CMs (iPSC-CMs; iCell cardiomyocytes) (Cellular Dynamic International, USA) were added and cryopreserved in a vial in liquid nitrogen. For cell culture, vials containing 1.5 × 10^6^ cardiomyocytes were thawed by immersing and shaking the cryovial in a 37 °C water bath. The thawed cardiomyocytes were rapidly transferred to a 15 mL centrifuge tube and diluted with 10 mL of ice-cold plating medium (Cellular Dynamic International, USA). The cells were collected by centrifugation at 1000 rpm and resuspended in 1 mL of plating medium. After thawing, 5 × 10^4^ cells were seeded in each well of the IDE device, and the device was fixed on the recording system and maintained in an incubator at 37 °C and 5% CO_2._ The culture medium was refreshed every 48 h. Impedance measurements were taken continuously after cardiomyocytes were seeded in the IDE device. Moreover, by observing the cells, cardiomyocyte seeding and viability were validated, as shown in Supplementary Video S1.

### Drug assay

Drug assays were performed when the rhythmic beating profiles of the cardiomyocytes became stable. For this study, three typical hERG K^+^ channel inhibitors, namely, astemizole (Sigma-Aldrich, USA), droperidol (Sigma-Aldrich, USA), and sertindole, were applied at various doses (16 nM, 80 nM, 400 nM, 2 μM, and 10 μM) to treat the cardiomyocytes. These drugs were prepared in dimethylsulfoxide (DMSO, Sigma-Aldrich, USA) and PBS and then prewarmed in a 37 °C water bath before being added to the culture wells. Mechanical beating signals were recorded before drug treatment as controls.

### Data analysis

Signal processing was performed with customized LabVIEW software. All statistical analyses were performed using Prism 8.0 (GraphPad Software Inc., USA) or Office Excel 2016 (Microsoft, USA). All results are presented as the mean ± standard deviation (SD). The data were analyzed by unpaired Student’s *t*-test, and differences between groups were considered statistically significant when *P* < 0.05.

## Results and discussion

### Characterization and optimization of cardiomyocyte mechanical beating templates

**Fig. 2:**
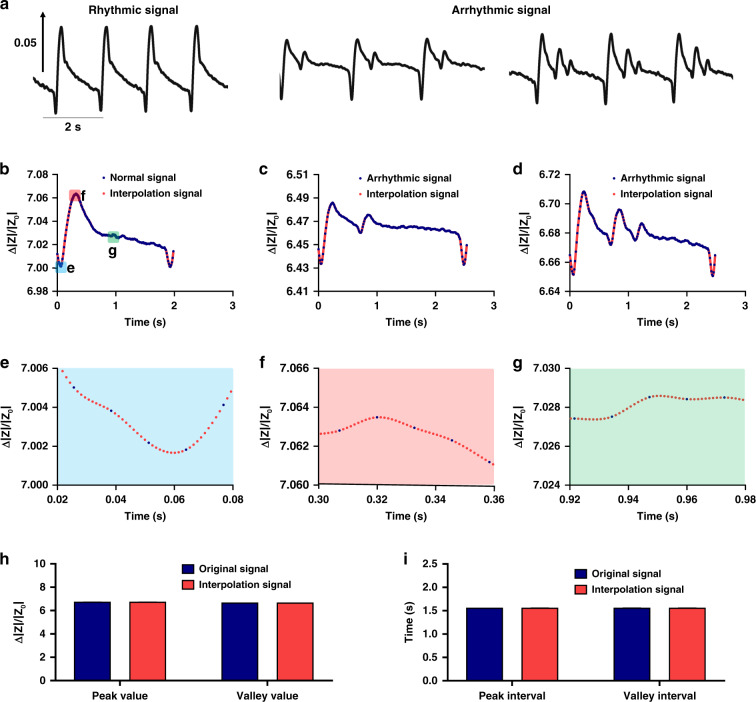
Characterization and optimization of cardiomyocyte mechanical beating templates. **a** Typical mechanical beating signals of cardiomyocytes recorded by the biosensing system. The left panel shows rhythmic signals, and the right panel shows arrhythmic signals under astemizole treatment with two peaks and three peaks. **b**–**d** Comparison of rhythmic and arrhythmic mechanical beating signals before and after spline interpolation. **e**–**f** Close-up view of typical features of the mechanical beating signals in (**b**). New points were added to the original signals by spline interpolation. **h** Comparison of signal peak and valley values before and after spline interpolation. The peak and valley values presented no significant difference. *n* = 3 from different signal segments. **i** Comparison of signal peak and valley intervals before and after spline interpolation. The peak and valley intervals presented no significant difference. *n* = 3 from different signal segments

To perform ATM, templates of cardiomyocyte mechanical beating were characterized and optimized. Human iPSC-CMs presented a mature and stable status after culture for 5 days (Supplementary Fig. S1). As shown in the left panel of Fig. 2a, the rhythmic mechanical beating signals of CMs were recorded by the IDE-based impedance measuring system at a high amplitude. Furthermore, to establish an arrhythmic model of cardiomyocytes, astemizole (a hERG K^+^ channel inhibitor^36^) was administered to the cells. Based on the sensitivity of human iPSC-CMs, arrhythmic mechanical beating will occur after astemizole treatment, and signal profiles with two and three positive peaks are typical arrhythmic signals. Arrhythmic mechanical beating is induced by hERG K^+^ channel blockade, which leads to the deactivation delay of Ca^2+^ channels, and the later Ca^2+^ inflow induces extramechanical beating of cardiomyocytes^9^. Conventionally, the measuring rate of impedance data are low due to the multichannel synchronized recording and high working frequency (10–100 kHz) of IDE devices, so the temporal resolution of signals is usually low, as shown by the blue dots in Fig. 2b–d. To improve the details of the signal and precisely determine the feature points, spline interpolation was employed to reconstitute the beating signals (red dots in Fig. 2b–d). The temporal resolution was improved from 12.8 to 1.28 ms by interpolating nine extra fitting points between original neighbor points without distorting the signal profiles, and the reconstituted signals were subjected to precise feature point detection (e.g., peak and valley) (Fig. 2e–g). After spline interpolation, the basic parameters were extracted from the interpolation signals. Compared with the parameters of the original signals, those of the interpolation signals were not significantly different. The mean peak and valley values remained at 6.758 and 6.687, respectively (Fig. 2h), while the mean peak and valley intervals remained at 1.563 and 1.563 s, respectively (Fig. 2i). Therefore, the beating signals were effectively optimized by spine interpolation to improve the temporal resolution of the signals without changing their profiles or key feature parameters.

**Fig. 3:**
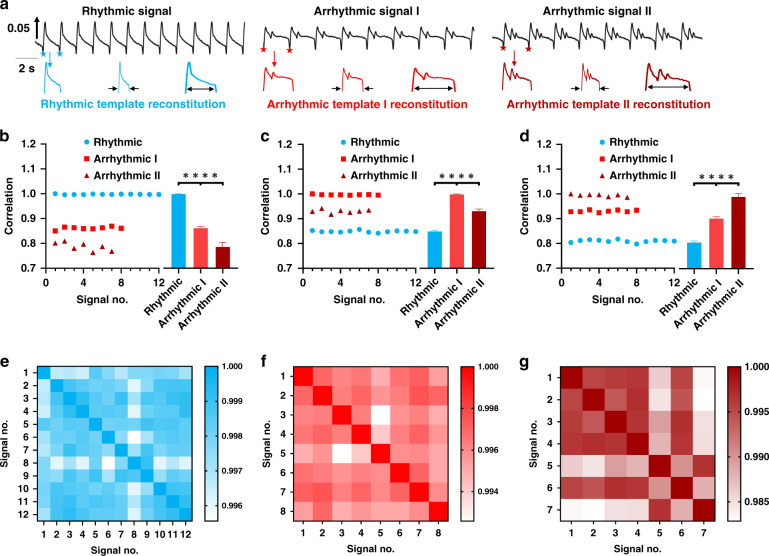
Characterization of random rhythmic and arrhythmic templates for mechanical beating recognition under 80 nM astemizole treatment in the short term. **a** Extraction and reconstitution of one rhythmic template and two arrhythmic templates. Templates were randomly extracted from typical mechanical beating signals by valley detection and were then reconstituted to match the data length of the target signals by spline interpolation and resampling. **b**–**c** Matching of the templates with the typical signals in (**a**) by correlation analysis. Templates and signals with similar profiles showed a high correlation (>0.98) in the short term, while templates and signals with different profiles showed a low correlation (<0.94). The correlation (<0.9) between the rhythmic template and arrhythmic signals was lower than that between the arrhythmic template and arrhythmic signals (>0.9). *n* ≥ 21 from three data segments; ****p < 0.0001. **e**–**g** Universality test of the random mechanical beating templates by analysis of the correlation between similar signals in the short term. The correlation between the rhythmic template and rhythmic signals was over 0.995. The correlation between arrhythmic template I with two positive peaks and arrhythmic signals I was over 0.992. The correlation between the arrhythmic template II with three positive peaks and arrhythmic signals II was over 0.982

After the original signals were optimized by spline interpolation, each complete beating signal could be defined and extracted by valley detection. As shown in Fig. 3a, the templates were randomly extracted from random signal segments. Typical rhythmic and arrhythmic templates were collected from corresponding signal segments as the basic matching templates. Based on these templates of mechanical beating, ATM was performed by analyzing the correlation between the template and each complete beating signal. However, the template signals seldom had the same data length as the target signal, so it was necessary to dynamically adjust the data length of the template signal to match the data length of the target signal for correlation analysis. The data length of the template signal was first prolonged by spline interpolation to a multiple of the data length of the target signal, and the data length of the template signal was then resampled to be the same as that of the target signal. To verify the specific recognition ability, the first complete beating signal was employed as the template to analyze the correlation of the signal with other complete signals from these three signal segments. As shown in Fig. 3b, the correlation between the rhythmic template and match rhythmic signal was significantly higher than that between the rhythmic template and the mismatched arrhythmic signal (rhythmic template vs. rhythmic signal: 0.9977 ± 0.0011; rhythmic template vs. arrhythmic signal I: 0.8613 ± 0.0053; rhythmic template vs. arrhythmic signal II: 0.7867 ± 0.0160). Figure 3c, d also presents similar results: the correlation between the matched template signal and target signal was significantly higher than that of the mismatched pair (arrhythmic template I vs. rhythmic signal: 0.8487 ± 0.0038; arrhythmic template I vs. arrhythmic signal I: 0.9967 ± 0.0014; arrhythmic template I vs. arrhythmic signal II: 0.9303 ± 0.0069; arrhythmic template II vs. rhythmic signal: 0.8094 ± 0.0051; arrhythmic template II vs. arrhythmic signal I: 0.9304 ± 0.0038; arrhythmic template II vs. arrhythmic signal II: 0.9942 ± 0.0046). To further test the universality of the template signal, each complete signal was extracted as a template, and the correlation of each of these signals with the other complete signals in the same segment was assessed. According to the correlation analysis patterns in Fig. 3e–g, the correlation between template signals and their similar signals in the short term was high (>0.982). To further determine the optimal threshold and prove the universality of the template selection method, the rhythmic profile and arrhythmic profile were traversed by a randomly selected rhythmic template. The correlation between rhythmic profiles is shown in Supplementary Fig. S3a. All of the correlation coefficients were larger than 0.94 except for one (0.939604). In addition, the correlation coefficient between a group arrhythmic profile and the rhythmic template was calculated and is shown in Supplementary Fig. S3b. The maximum value of the correlation was 0.935967, and over 95% of the correction coefficients were <0.9. Thus, 0.94 was selected as the threshold value of the rhythmic template. To further verify the universality of the template selection method, the two paired profiles with the lowest correlation coefficient in Supplementary Fig. S3a were traversed through a set of rhythmic templates, as shown in Supplementary Fig. S3c, d. The minimum correlation (0.94399) was larger than the threshold (0.94). By the same method, a threshold value of 0.9 was selected for the arrhythmic template.

Moreover, the necessity of matching the data length between the template and target signal was further determined. Supplementary Fig. S2a–c shows the comparison of correlations between length-matched and length-mismatched templates. It was obvious that length-matched templates presented a higher correlation with the target signals than length-mismatched templates. In addition, the recognition accuracy of the length-matched templates was more stable than and superior to that of the length-mismatched templates. As shown in Supplementary Fig. S2d–f, the length-mismatched random templates displayed a lower correlation with the target signals than length-matched templates (rhythmic: 0.957 vs. 0.995; arrhythmic I: 0.878 vs. 0.992; arrhythmic II: 0.863 vs. 0.982). It was demonstrated that spline interpolation was necessary to significantly improve the correlation between the template and similar beating signals for ATM.

### Arrhythmia recognition by ATM under treatment with a hERG K^+^ channel inhibitor

**Fig. 4:**
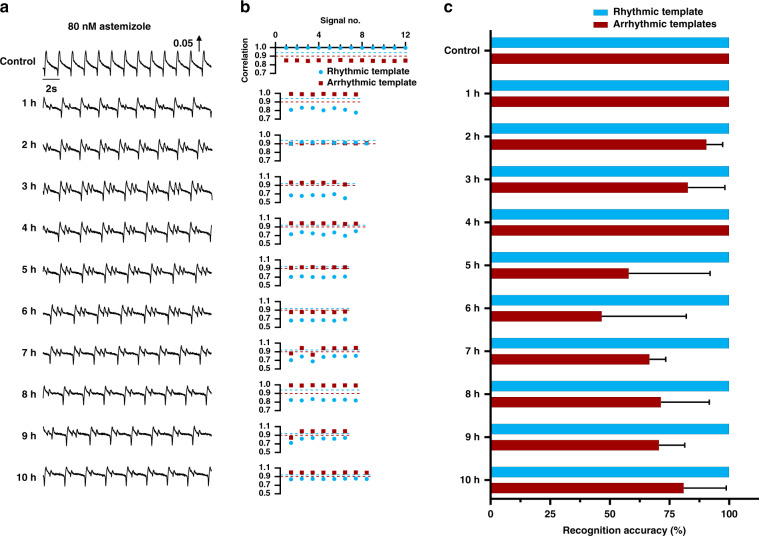
Long-term arrhythmia recognition ability of ATM under hERG K^+^ channel inhibitor treatment. **a** Typical mechanical beating signals under 80 nM astemizole treatment, which were randomly extracted every hour after drug treatment. The signals indicate the arrhythmia profiles after astemizole treatment for 10 h. **b** Correlation analysis of templates and target mechanical beating signals in (**a**). Based on the short-term analysis results, the correlation threshold for rhythmic template matching was set at 0.94 (blue dashed line) the signal was considered rhythmic if the correlation coefficient was over 0.94, and the signal was considered arrhythmic if the correlation coefficient was below 0.94. Furthermore, the correlation threshold for arrhythmic template matching was set at 0.9 (red dashed line); the signal was considered arrhythmic if the correlation coefficient was over 0.9, and the signal was considered rhythmic if the correlation coefficient was below 0.9. Taking the mixed arrhythmic profiles (two and three positive peaks) of mechanical beating signals into account, arrhythmic multitemplates were simultaneously applied for target arrhythmic signal recognition, and the correlation coefficient was used to determine the signal type. **c** Recognition accuracy by rhythmic and arrhythmic templates. The recognition accuracy based on the rhythmic template reached 100% in the long term, while the recognition accuracy based on the arrhythmic multitemplates was low and unstable, ranging from 46.6 ± 35.39% to 100%. *n* ≥ 18 from three different signal segments

To further verify the performance of ATM, the same rhythmic and arrhythmic templates were employed to recognize arrhythmic signals under 80 nm astemizole treatment in the long term. Signal segments were randomly collected each hour after astemizole treatment, as shown in Fig. 4a. Compared with those in the control group, the beating signal profiles in the treatment group varied and included two-peak, three-peak, and mixed patterns. The template signals were divided into two categories: rhythmic templates and arrhythmic multitemplates. The arrhythmic templates contained two-peak and three-peak profiles. Based on the short-term correlation analysis of template signals (Fig. 3b–d), the rhythmic template was employed to recognize the arrhythmic signal when the correlation coefficient was <0.94. However, the arrhythmic multitemplates were used to simultaneously recognize the arrhythmic signal when the correlation coefficient was >0.9. To improve the recognition accuracy, the higher correlation coefficient for the two arrhythmic templates was selected as the output result. The correlations between individual beating signals and rhythmic and arrhythmic templates are shown in Fig. 4b. The rhythmic template was able to not only recognize all the rhythmic beating signals but also accurately find all the arrhythmic beating signals. The individual correlation analysis (blue circle) presented high specificity due to the rhythmic threshold (0.94) selected for random signal segments, which were randomly extracted each hour. However, the arrhythmic multitemplates failed to recognize all the arrhythmic beating signals. As shown in Fig. 4c, the recognition accuracy of the rhythmic template reached 100% (total recognition rate of 100% with no mismatched result among 242 randomly extracted signals) for three random signal segments extracted each hour, while the recognition accuracy of the arrhythmic template was low and unstable, ranging from 46.62 ± 35.39% to 100% (total recognition rate of 82.2% with 43 mismatched results among 242 randomly extracted signals).

**Fig. 5:**
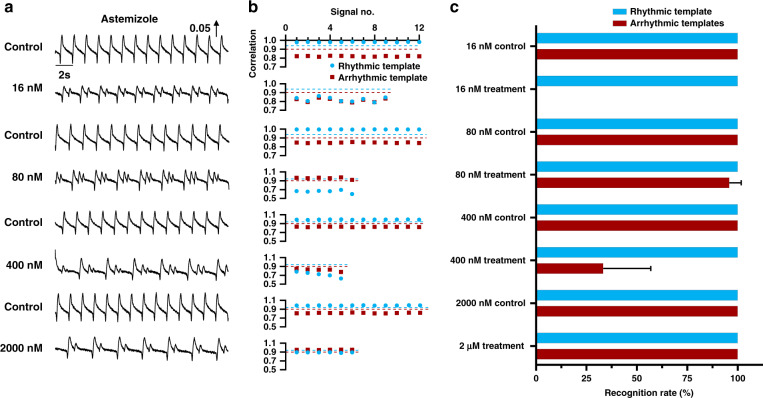
Arrhythmia recognition by ATM under treatment with different doses of the same hERG K^+^ channel inhibitor. **a** Typical mechanical beating signals before and after 16, 80, 400, or 2000 nM astemizole treatment. The signals presented arrhythmic profiles after astemizole treatment at different doses. **b** Correlation analysis between the templates and target mechanical beating signals in (**a**). The correlation threshold for rhythmic template matching was set at 0.94 (blue dashed line), while the correlation threshold for arrhythmic template matching was set at 0.9 (red dashed line). Arrhythmic multitemplates were simultaneously applied for target arrhythmic signal recognition, and the correlation coefficient was used to determine the signal type. **c** Recognition accuracy by rhythmic and arrhythmic templates. The recognition accuracy based on the rhythmic template reached 100% under astemizole treatment at different doses, while the recognition accuracy based on the arrhythmic multitemplates as low and unstable under treatment with astemizole at different doses, ranging from 0% to 100%. *n* ≥ 15 from three different signal segments

In addition to arrhythmic recognition under long-term drug treatment, the performance of ATM was further verified in the presence of astemizole at different doses. The templates previously extracted under 80 nM astemizole treatment remained constant and were applied to recognize random arrhythmic beating signals in the presence of 16–2000 nM astemizole (Fig. 5a). The rhythmic template was used to recognize arrhythmic beating signals with a correlation threshold of 0.94, and the arrhythmic multitemplates were used to recognize arrhythmic beating signals with a correlation threshold of 0.9. As shown in Fig. 5b, the rhythmic template was successful in recognizing all the rhythmic and arrhythmic beating signals from the random signal segments under treatment at different doses, even if the template was extracted from a different signal segment. However, arrhythmic multitemplates still failed to recognize arrhythmic beating signals. The results of statistical analysis of ATM recognition for arrhythmic beating signals under treatment at different doses is presented in Fig. 5c. The recognition accuracy of the rhythmic template reached 100% (total recognition rate of 100% with no mismatched result among 184 randomly extracted signals) for three random signal segments extracted from each dose, while the recognition accuracy of the arrhythmic template was low and unstable, ranging from 0 to 100% (total recognition rate of 77.7% with 41 mismatched results among 184 randomly extracted signals). The recognition function of ATM by rhythmic templates or arrhythmic multitemplates was determined as the correlation between the templates and the target signal. Although the target beating signal presented similar profiles as the arrhythmic template, the positions of their multiple peaks matched with those in the templates, and this could not be effectively improved by adjusting the data length, so the correlation was relatively low. Unlike recognition by arrhythmic multitemplates, recognition by the length-matched rhythmic template could be employed to find mismatched arrhythmic beating signals; this method is sensitive to sensing arrhythmic beating signals with small differences via correlation analysis. Therefore, it was demonstrated that the rhythmic template can accurately recognize signal types under treatment with different doses of the same hERG K^+^ channel inhibitor in the long term.

### Arrhythmia recognition by ATM under treatment with different hERG K^+^ channel inhibitors

**Fig. 6:**
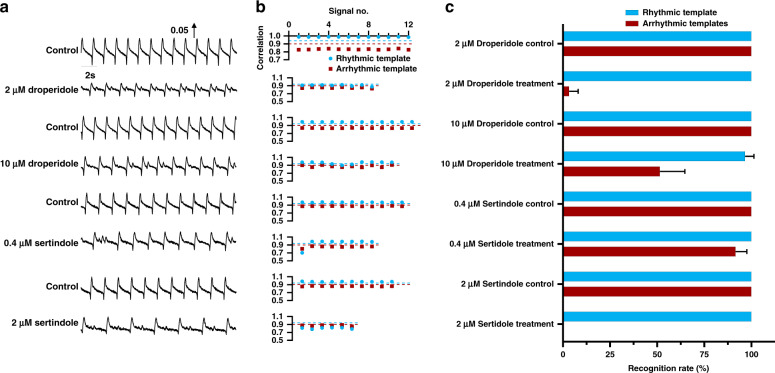
Arrhythmia recognition by ATM under treatment with other hERG K^+^ channel inhibitors at different doses. **a** Typical mechanical beating signals before and after droperidol (2 and 10 μM) and sertindole (0.4 and 2 μM) treatment. The signals present the arrhythmia profiles after droperidol and sertindole treatment at different doses. **b** Correlation analysis of the templates and target mechanical beating signals in (**a**). The correlation threshold for rhythmic template matching was set at 0.94 (blue dashed line), while the correlation threshold for arrhythmic template matching was set at 0.9 (red dashed line). Arrhythmic multitemplates were simultaneously applied for target arrhythmic signal recognition, and a the correlation coefficient was used to determine the signal type. **c** Recognition accuracy by rhythmic and arrhythmic templates. The recognition accuracy based on the rhythmic template reached over 96.67 ± 4.71% under droperidol and sertindole treatment at different doses, while the recognition accuracy based on the arrhythmic multitemplates was low and unstable under treatment at different doses, ranging from 3.33 ± 4.71% to 100%. *n* ≥ 15 from three different signal segments

To further test the universality of ATM, other hERG K^+^ channel inhibitors (droperidol^37^ and sertindole^38^) were applied, and the performance of the templates was determined. Figure 6a shows the typical beating signal segments before and after hERG K^+^ channel inhibitor treatment at different doses. Compared with the rhythmic beating signals, the arrhythmic beating signals presented various profiles mixed with normal beating signals in the control group. The templates previously obtained under astemizole treatment were used to recognize arrythmia induced by different drugs. As shown in Fig. 6b, the rhythmic template was still successful in recognizing all the rhythmic and most arrhythmic beating signals from the random signal segments under droperidol and sertindole treatment at different doses. Furthermore, the arrhythmic multitemplates did not match the many arrhythmic beating signals. After 2 μM droperidol treatment, a poor correlation with the arrhythmic templates but a high correlation with the rhythmic template was found. This poor correlation may have originated from the offset of peaks between the templates and the rhythmic signals, as shown in Supplementary Fig. S5. Figure 6c shows the results of statistical analysis of ATM recognition for arrhythmic beating signals under treatment at different doses. The recognition accuracy of the rhythmic template was over 96.67 ± 4.71% (total recognition rate of 99.14% with only two mismatched results among 233 randomly extracted signals) for three random signal segments extracted under each condition. However, the recognition accuracy of the arrhythmic template was low and unstable, ranging from 3.33 ± 4.71% to 100% (total recognition rate of 74.25% with 60 mismatched results among 233 randomly extracted signals). Moreover, the arrhythmic recognition performance of ATM for the clean hERG blocker E-4031 is shown in Supplementary Fig. S6. The recognition accuracy of the rhythmic template was over 98.81 ± 2.06% for three random signal segments extracted under each condition. However, the recognition accuracy of the arrhythmic template was low and unstable, ranging from 45.56 ± 38.52% to 77.53 ± 10.06%. The arrhythmic multitemplates were often unable to recognize beating profiles with unobvious/low-amplitude arrhythmia in addition and those in which the peak was shifted. Unlike the arrhythmic multitemplates, the rhythmic template was able to recognize most beating signal types with fewer mismatched cases, and the recognition accuracy of the rhythmic template for arrhythmic beating signals, which are easily ignored by examination by the naked eye or signal processing, was much better than that of the arrhythmic multitemplates.

## Conclusion

In this study, an ATM model of cardiomyocyte beating induced by hERG K^+^ channel inhibitors was established based on a human iPSC-CM biosensing system, which can collect the mechanical beating signals of cardiomyocytes by IDEs and an impedance measurement technique. By extracting rhythmic templates and adjusting them via spline interpolation, drug-induced arrhythmic beating signals could be accurately recognized by the rhythmic template. The rhythmic template performed well and could not only accurately recognize the arrhythmic beating signals induced by hERG K^+^ channel inhibitors at different doses in the long term but could also recognize the arrhythmic beating induced by different hERG K^+^ inhibitors. Combining a high-specificity cardiomyocyte-based biosensor and advanced signal processing, this ATM method based on a cardiomyocyte beating model may be a sensitive, efficient, and precise approach for the safety screening of cardiac drugs and may be a promising tool for protecting human health in the fields of cardiology and pharmacology.

## Supplementary information

Supplementary Information

Supplementary Information video
