# Supplementary material for: Recognition of high-specificity hERG K+ channel inhibitor-induced arrhythmia in cardiomyocytes by automated template matching
Source: Microsyst Nanoeng. 2021 Mar 16;7:24. doi: 10.1038/s41378-021-00251-4 (PMC8433465; doi:10.1038/s41378-021-00251-4)
Supplement: Supplementary file 1 — Supplementary Information [file 41378_2021_251_MOESM1_ESM.docx]

**Supplementary**

**High-specificity hERG K+ channel inhibitor induced arrhythmia recognition of cardiomyocytes by automated template match**

***Hao Wang^a+^, Hongbo Li^a+^, Xinwei Wei^b+^, Tao Zhang^a^,*** ***Yuting Xiang^c^ Jiaru Fang^a^, Peiran Wu^a^, Xi Xie^a^*, Ping Wang^b,d^*, Ning Hu^a,d^****

^a^The First Affiliated Hospital of Sun Yat-sen University, School of Electronics and Information Technology, State Key Laboratory of Optoelectronic Materials and Technologies, Guangdong Province Key Laboratory of Display Material and Technology, Sun Yat-sen University, Guangzhou 510006, China.

^b^Biosensor National Special Laboratory, Key Laboratory of Biomedical Engineering of Ministry of Education, Department of Biomedical Engineering, Zhejiang University, Hangzhou 310027, China.

^c^The Sixth Affiliated Hospital of Sun Yat-sen University, Guangzhou 510655, China.

^+^These authors contribute equally to this work.

^d^State Key Laboratory of Transducer Technology, Chinese Academy of Sciences, Shanghai 200050, China.

*To whom correspondence may be addressed. Corresponding to: Ning Hu, [huning3@mail.sysu.edu.cn](mailto:huning3@mail.sysu.edu.cn); Ping Wang, cnpwang@zju.edu.cn; Xi Xie, xiexi27@mail.sysu.edu.cn

**
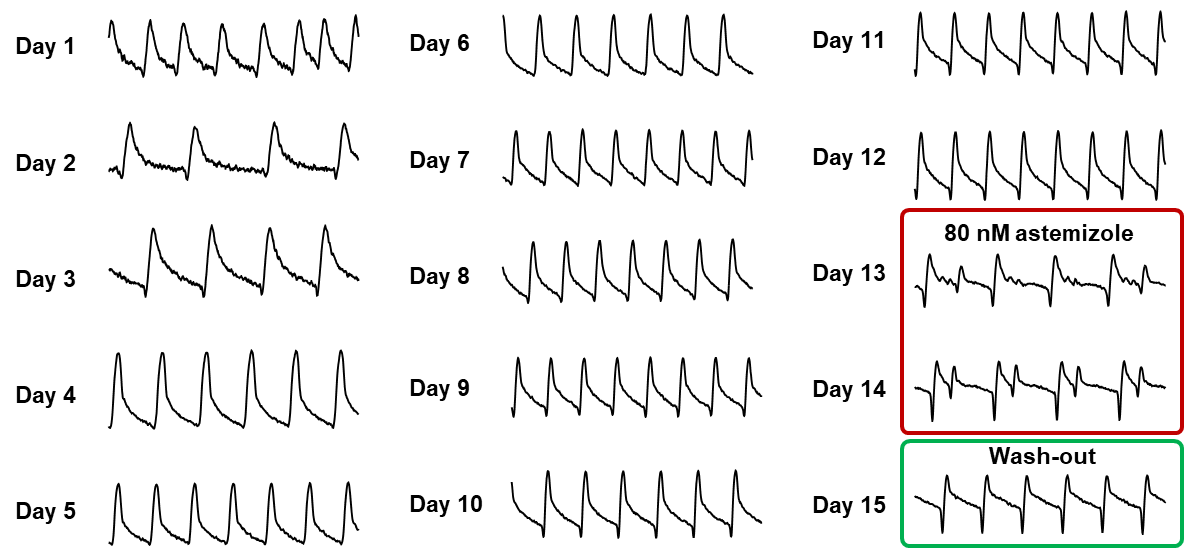
**

**Supplementary Figure S1. Typical native and drug-induced mechanical beating signals of cardiomyocytes during long-term culture. Cardiomyocyte presents the stable and mature status after 5-day culture, and can last for a long period of time. hERG K^+^ channel inhibitor (astemizole) can induce the arrhythmic beating signals, and recover to normal status after refreshing the medium.**

**
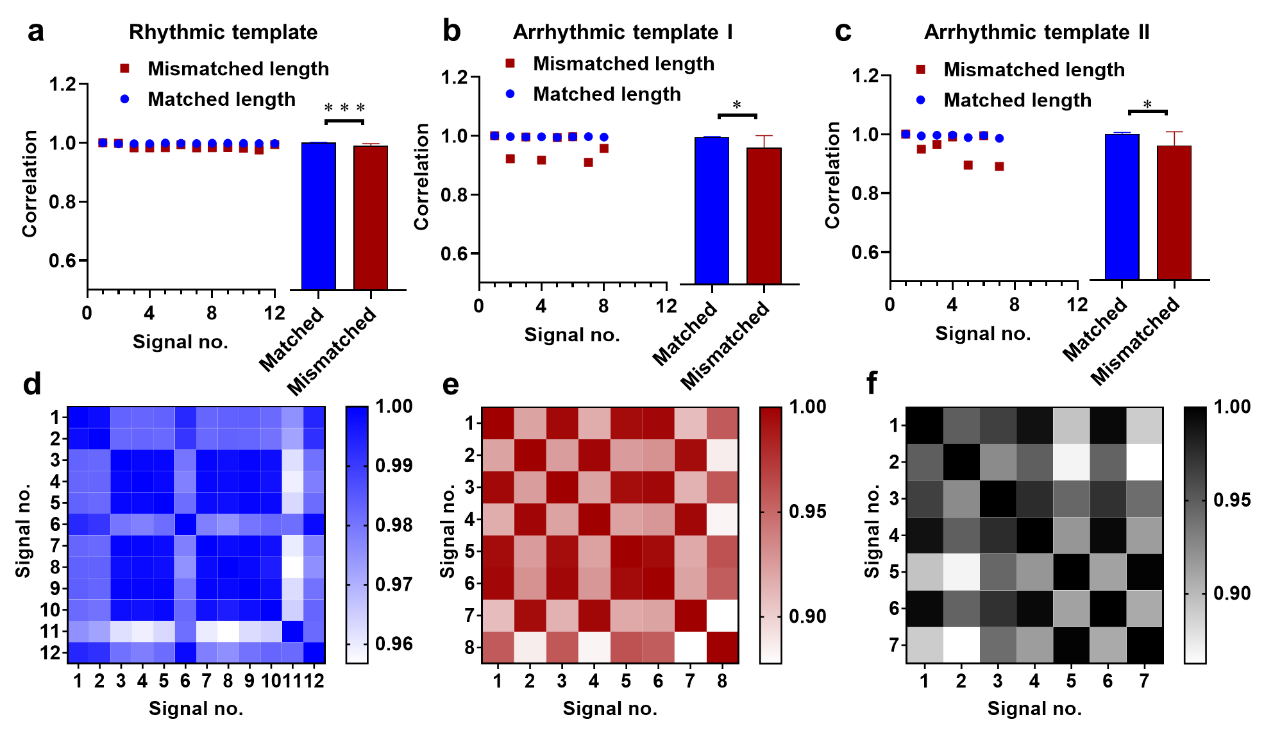
**

**Supplementary Figure S2. Comparison of the correlation analysis with and without spline interpolation. (a) Comparison of the correlation analysis of rhythmic template with matched length and mismatched length. (b) Comparison of the correlation analysis of arrhythmic template I with matched length and mismatched length. (c) Comparison of the correlation analysis of arrhythmic template II with matched length and mismatched length. (d-f) Universality test of random mechanical beating templates by analyzing the correlation with their similar signals in short term. The correlation between rhythmic template and rhythmic signals is over 0.957. The correlation arrhythmic template I with two positive peaks and arrhythmic signals I is over 0.878. The correlation arrhythmic template II with three positive peaks and arrhythmic signals II is over 0.863. The length-mismatched template showed low correlation with similar signals.**


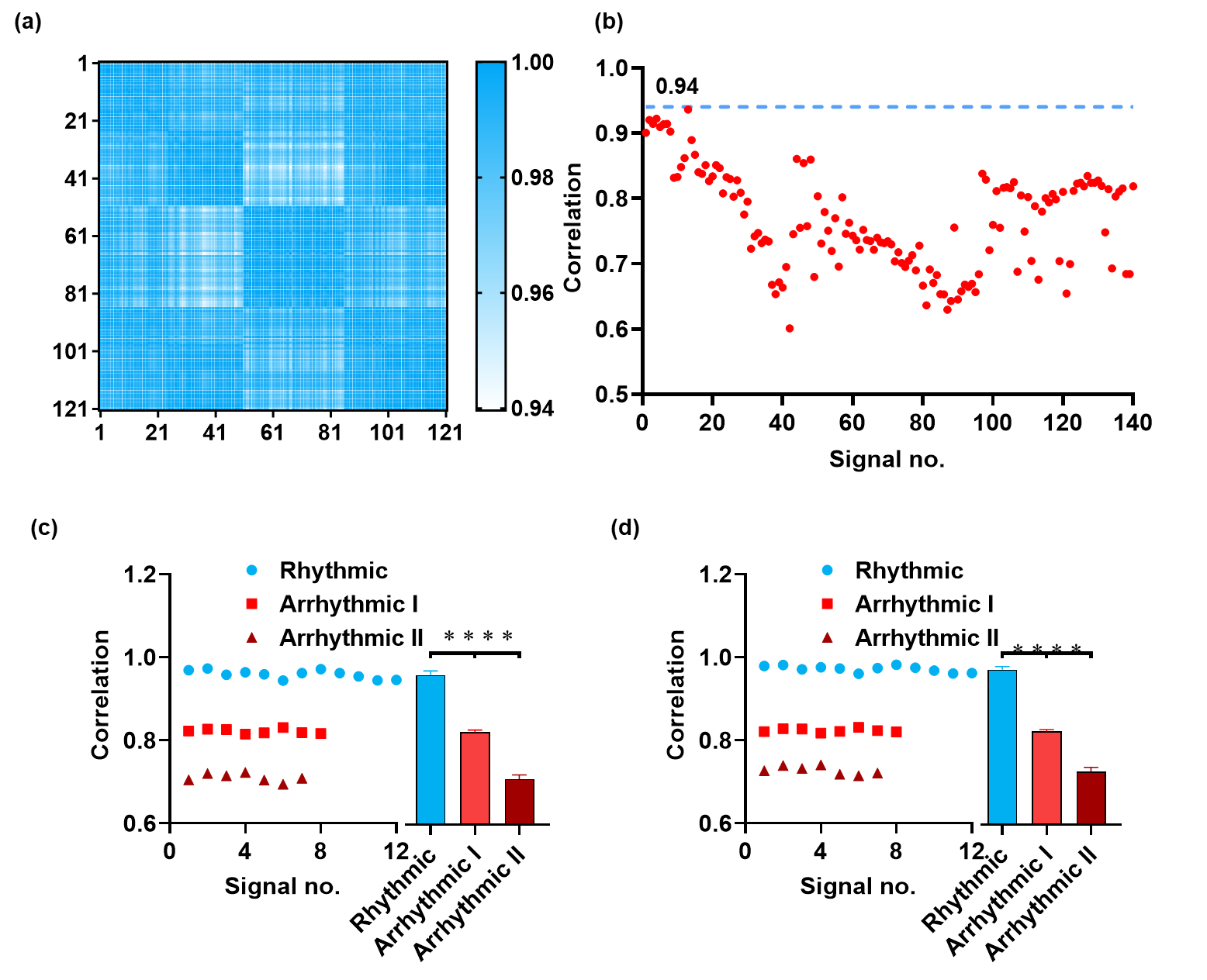


**Supplementary Figure S3.** Validation of the randomness of template selection and selection of the optimal threshold value. (a) The correlation coefficients between 121 rhythmic profiles. To find the optimal threshold value, the correlation coefficient between rhythmic profiles were calculated. The minimum correlation coefficients of two rhythmic is 0.939604. (b) The correlation coefficient of rhythmic template and arrhythmic data. (c - d) The correlation coefficient between two random rhythmic template and a random group of rhythmic data, which the rhythmic template is maximum difference in all rhythmic profile. The minimum correlation (0.94399) is larger than the threshold (0.94).


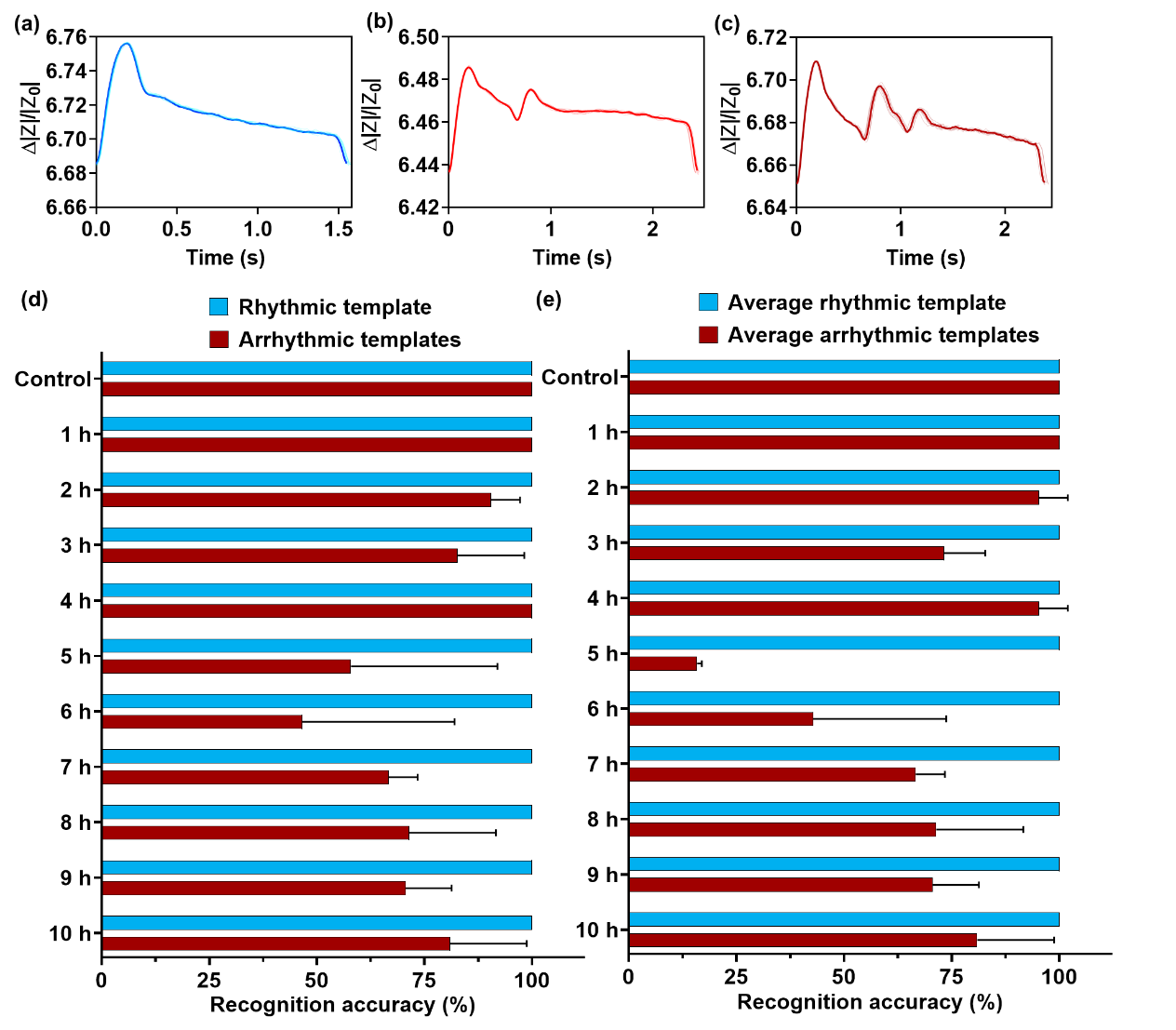


**Supplementary Figure S4.** The recognition performance comparison of random templates and average templates. (a) The superimposition of rhythmic profiles (light blue) and the average rhythmic profile (deep sky blue). (b) The superimposition of two-peak profiles (light red) and the average two-peak profile (red). (c) The superimposition of three-peak profiles (light red) and the average three-peak profile (darkred). (d) The long-term test using random rhythmic template. (e) The long-term test using average rhythmic template. In the 10 h after the addition of 80 nM astemizole, data of three groups were randomly extracted every hour. The result showed that the recognition accuracy of single rhythmic template and average rhythmic template were all reached 100%. Neither the single arrhythmic templates match nor the average arrhythmic templates match reaches 100% accuracy. In detail, In detail, two templates presents low recognition accuracy at 5h (single57.93647±34.02537% and average 15.87303±1.12241%). The difference may originate from the offset of peaks between the arrhythmic templates and the average arrhythmic templates, resulting in the decrement of correlation coefficients.


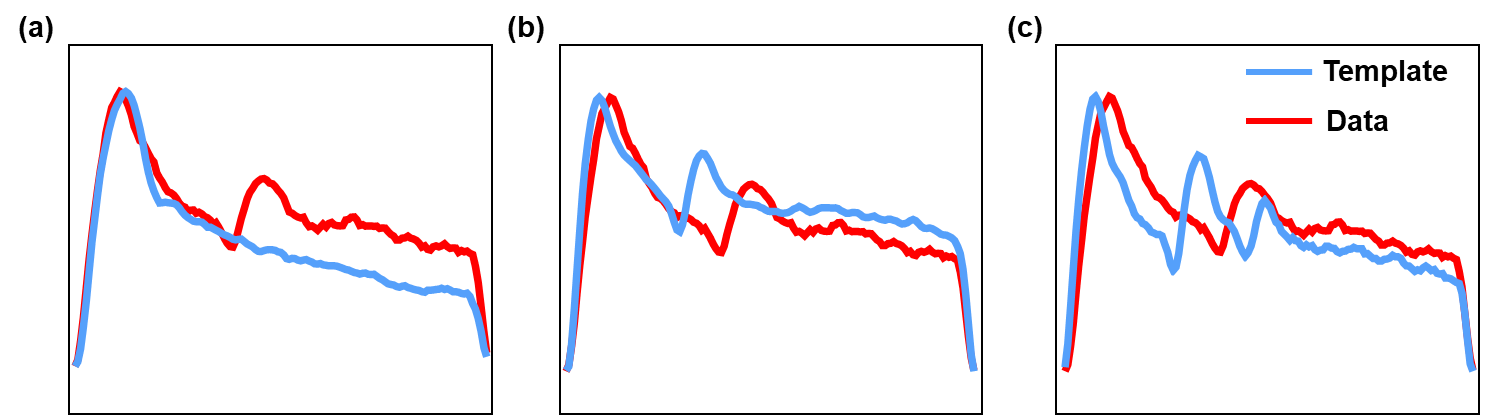


**Supplementary Figure S5.** Comparison of the arrhythmia profiles induced by 2 μM Droperidole with three type templates: (a) rhythmic template, (b) two-peak template, (c) three-peak template. Templates were in blue and 2 μM Droperidole induced arrhythmia was in red. The mismatch of peaks between the arrhythmic profile and the arrhythmic templates caused the lower correlation coefficients than rhythmic template.

**
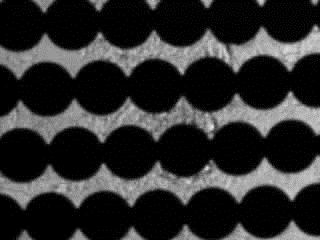
**

**Supplementary Video S1.** The cardiomyocyte on the interdigital electrode. Observation of the cell status on the interdigital electrode to validate cardiomyocyte seeding and viability.


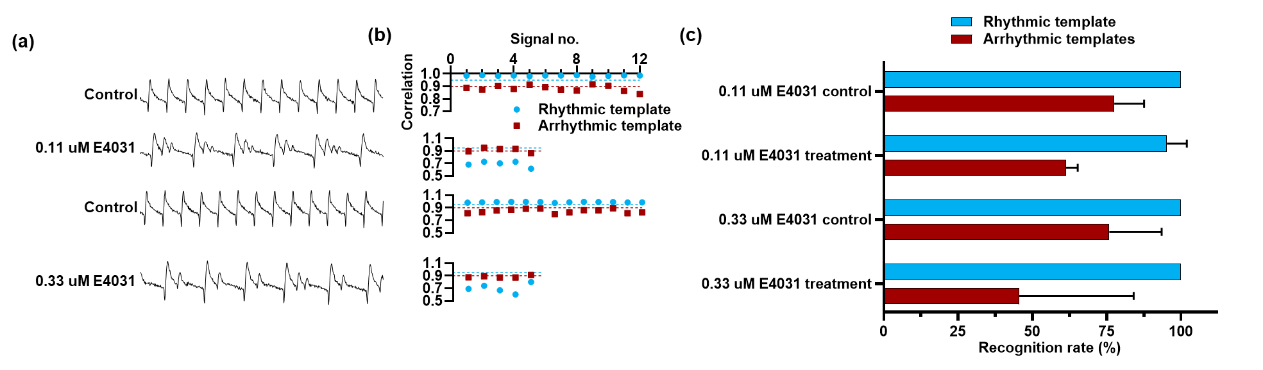


**Supplementary Figure S6.** Arrhythmia recognition of automated template match (ATM) under E-4031 hERG K^+^ channel inhibitor treatment. (a) Typical mechanical beating signals before and after E-4031 (0.11 and 0.33 μM) treatment. The signals present the arrhythmia profiles after the E-4031 treatment in different doses. (b) Correlation analysis of templates and target mechanical beating signals in (a). The correlation threshold of rhythmic template match is set at 0.94 (blue dashed line), which the correlation threshold of arrhythmic template match is set at 0.9 (red dashed line). The arrhythmic multi-templates are simultaneously applied for target arrhythmic signal recognition, and the large correlation is remained to determine the signal type. (c) Recognition accuracy by rhythmic and arrhythmic templates. The recognition accuracy based on the rhythmic template reaches over 98.81±2.06% under E-4031 with different doses, while the recognition accuracy based on the arrhythmic multi-templates is low and unstable at different doses ranging for 45.56±38.52% to 77.53±10.06%. n≥15 from three different signal segments.
